# Histidine Metabolism and IGPD Play a Key Role in Cefquinome Inhibiting Biofilm Formation of *Staphylococcus xylosus*

**DOI:** 10.3389/fmicb.2018.00665

**Published:** 2018-04-05

**Authors:** Yong-hui Zhou, Chang-geng Xu, Yan-bei Yang, Xiao-xu Xing, Xin Liu, Qian-wei Qu, Wen-ya Ding, God’spower Bello-Onaghise, Yan-hua Li

**Affiliations:** ^1^College of Veterinary Medicine, Northeast Agricultural University, Harbin, China; ^2^Heilongjiang Key Laboratory for Animal Disease Control and Pharmaceutical Development, Harbin, China

**Keywords:** *S. xylosus*, biofilm, cefquinome, iTRAQ, histidine metabolism, IGPD

## Abstract

*Staphylococcus xylosus* (*S. xylosus*) is an AT-rich and coagulase-negative *Staphylococcus* (CNS). It is normally regarded as non-pathogenic, however, recent studies have demonstrated that it is related to human opportunistic infections and bovine mastitis. In addition, *S. xylosus* strains have the ability to form biofilm. Biofilms are also involved in chronic infections and antibiotic resistance, there are only a few reports about cefquinome inhibiting *S. xylosus* biofilm formation and the protein targets of cefquinome. In our study, we found that sub-MICs of cefquinome were sufficient to inhibit biofilm formation. To investigate the potential protein targets of cefquinome, we used iTRAQ for the analyses of cells at two different conditions: 1/2-MIC (0.125 μg/mL) cefquinome treatment and no treatment. Using iTRAQ technique and KEGG database analysis, we found that proteins differently expression in histidine metabolism pathway may play a role in the process by which 1/2-MIC (0.125 μg/mL) cefquinome inhibits *S. xylosus* biofilm formation. Interestingly, we found a sharply down-regulated enzyme [A0A068E9J3 imidazoleglycerol-phosphate dehydratase (IGPD)] involved in histidine metabolism pathway in cefquinome-treated cells. We demonstrated the important role of IGPD in sub-MICs cefquinome inhibiting biofilm formation of *S. xylosus* by gene (*hisB*) knockout, IGPD enzyme activity and histidine content assays. Thus, our data sheds light on important role of histidine metabolism in *S. xylosus* biofilm formation; especially, IGPD involved in histidine metabolism might play a crucial role in sub-MICs cefquinome inhibition of biofilm formation of *S. xylosus*, and we propose IGPD as an attractive protein target of cefquinome.

## Introduction

*Staphylococcus xylosus* is a CNS found on the skin of mammals and in food. This AT-rich gram-positive bacteria is normally regarded as non-pathogenic. However, recent studies have demonstrated that *S. xylosus* might be involved in human opportunistic infections and bovine mastitis (Akhaddar et al., 2010; Rumi et al., 2013; Tan et al., 2014). In addition, *S. xylosus* strains have the ability to form biofilm which could lead to chronic infections and antibiotics resistance (Parsek and Singh, 2003; Planchon et al., 2006).

Bacterial biofilm formation is a result of several complex molecular mechanisms involving a wide range of proteins that are thought to play major roles in cell adhesion, maturation, signaling, etc. (Sauer, 2003; Latasa et al., 2006; Beloin et al., 2008; Gaddy and Actis, 2009). It has been shown that metabolism of nutrients has a great influence on biofilm formation (Planchon et al., 2006). Of interest are pathways involved in nitrogen metabolism, amino acids metabolism, and histidine synthesis pathway, all of which has been reported to have a major impact on the biofilm formation of *S. xylosus* (Planchon et al., 2009; Xu et al., 2017). Indeed, the L-histidine synthesis pathway involved in nitrogen metabolism has been shown to be involved in biofilm formation (Kulis-Horn et al., 2014; Zeidan et al., 2014). L-histidine is one amongst the 21 proteinogenic amino acids that can be synthesized *de novo* in lower eukaryotes and prokaryotes (Dietl et al., 2016). Histidine biosynthesis comprises of 10 enzymatic reactions involving proteins encoded from seven bacterial genes, and are highly conserved in lower eukaryotes and prokaryotes. Imidazoleglycerol-phosphate dehydratase (IGPD) catalyzes the sixth step in the histidine biosynthesis pathway, and is the first enzyme exclusively dedicated to histidine biosynthesis in bacteria (Alifano et al., 1996). IGPD has been widely studied as a target for herbicides for years (Ahangar et al., 2013); however, recent reports indicate the emerging role of IGPD in biofilm formation (Xu et al., 2017).

Previous studies have suggested that there is a relationship between some antimicrobial agents and biofilm (Majtan et al., 2008; Nucleo et al., 2009; Mishra et al., 2014; Zhao et al., 2015). Cefquinome, a fourth generation cephalosporin, is a broad-spectrum β-lactam antibiotic. Cefquinome is used to treat clinical mastitis, via the intramammary and parenteral routes and is licensed as a combination therapy for *E. coli* mastitis in the United Kingdom (Swinkels et al., 2013). Concurrent use of intramammary and parenteral cefquinome in clinical mastitis has also been evaluated (Swinkels et al., 2013). However, there is no report about cefquinome inhibiting *S. xylosus* biofilm formation and the protein targets of the cefquinome.

Biofilm is a complex process controlled by various factors. Many studies have analyzed the entire proteome of microorganisms using high-throughput proteomic tools to obtain a better understanding of factors involved in biofilm formation (Wang et al., 2012; Chen et al., 2014). Previous studies have investigated the sub-proteome analyses of planktonic and found the insights of the physiological and metabolic versatility. Planchon et al. (2009) gained insight into protein determinants of biofilm formation via comparative proteomic analysis of *S. xylosus* C2a strain. However, their study was mainly focused on differential expression of proteins in planktonic cells and biofilm cells. While their study provided insights into novel players in biofilm formation, further studies are required for a better understanding of biofilm formation that could lead to therapeutic designs for drug targets. In our study, we screened for the inhibitory levels of cefquinome against *S. xylosus* biofilm formation and utilized iTRAQ technology (Ow et al., 2009) to identify potential protein targets of cefquinome-mediated inhibition of biofilm formation. Our results indicate that sub-optimal levels of MIC of cefquinome are sufficient to inhibit biofilm formation. Interestingly, our proteomic data and *in vitro* assays indicate a key role for the histidine biosynthesis pathway enzyme IGPD in the biofilm formation of *S. xylosus*. Our study indicates IGPD as a cefquinome-target and proposes that IGPD could serve as an attractive target for the development of novel anti-biofilm drugs.

## Materials and Methods

### Growth Conditions

*Staphylococcus xylosus* strain cells of ATCC 700404 was grown overnight in Tryptic Soy Broth (TSB, Oxoid) media at 37°C with constant shaking.

### Minimum Inhibitory Concentration (MIC) Determination

Cefquinome was purchased from Qilu Animal Health Products Co., Ltd. (Jinan, China). The MIC of cefquinome was determined in TSB (Oxoid) using a broth dilution micromethod, according to the guidelines of Clinical and Laboratory Standards Institute (2003). Negative controls (wild-type *S. xylosus* ATCC700404 cells without cefquinome) and vehicle controls (culture media) were included. MIC was defined as the lowest concentration of cefquinome required for completely inhibiting microbial growth after 24 h of incubation. The experiments were performed in triplicate.

### Biofilm Formation

Biofilm formation was performed in 96-well microtiter plates, after which they were stained with the crystal violet method as previously described but with some modifications (Stepanovic et al., 2000; Sun et al., 2017). The overnight strains were grown in 5 ml TSB medium at 37°C. Then, dilute the cultures of cells at the concentration to 1 × 10^5^CFU/ml. Next, 200 μl of fresh TSB medium with different treatment suspensions were added to each well, and then incubated without shaking for 24 h at 37°C. Each well was rinsed thoroughly with 200 μl PBS three times to remove planktonic cells, the remaining attached bacteria were fixed with 200 μL 99% methanol (Guoyao Ltd., China) per well, and then the wells were left to dry. Following this, the biofilm was stained with 200 μl 0.1% crystal violet (Sularbao Ltd., Beijing, China) for 30 min at room temperature. After incubation, the remaining crystal violet was poured out, the wells were washed in the same manner, and the crystal violets in combination with biofilm were solubilized with 200 μl 33% (v/v) glacial acetic acid (Sularbao Ltd., Beijing, China). Finally, the sample absorbances were measured at 595 nm.

Different treatments: (1) Biofilm formation of wild-type *S. xylosus* ATCC700404 in the presence of sub-MIC cefquinome: Sub-MIC [1/2-MIC (0.125 μg/mL), 1/4-MIC (0.0625 μg/mL), and 1/8-MIC (0.03125 μg/mL)]; (2) Biofilm formation of wild-type *S. xylosus* ATCC700404 and mutant strains (inactivation of the *hisB* gene) with no treatments; (3) Biofilm formation of mutant strain (inactivation of the *hisB* gene) supplemented with histidine (0.5, 1, and 5 mM) treated; (4) Biofilm formation of mutant strain (inactivation of the *hisB* gene) supplemented with 1/2MIC (0.125 μg/mL) cefquinome treated and non-treated; and (5) Biofilm formation of wild-type *S. xylosus* ATCC700404 in the presence of 1/2-MIC cefquinome and supplemented with histidine (0.5, 1, and 5 mM) treated. All these five experiments were used the wild-type *S. xylosus* ATCC700404 strains with no treatment as control. The experiments were performed in triplicate.

### Scanning Electron Microscopy (SEM)

The biofilm structure of *S. xylosus* ATCC700404 was observed by SEM (Xu et al., 2017). Overnight cultures of *S. xylosus* ATCC700404 were diluted in sterile TSB (corresponding to 1 × 10^5^ CFU/ml). Then, the culture medium was supplemented sub-MIC of cefquinome, or without cefquinome (control), and 2 mL was added to wells of a 6-well microplate containing a 10 mm × 10 mm sterilized rough organic membrane (Mosutech Co., Ltd., Shanghai, China), respectively, on the bottom. After incubation without shaking for 24 h at 37°C, we took out organic membrane (Biofilms grow on it), medium and planktonic bacteria on the organic membrane were removed by washing with sterile PBS. The biofilms prepared for analysis as described by Xu et al. (2017).

### iTRAQ Analysis

Proteins were extracted at two different conditions [1/2-MIC (0.125 μg/mL) of cefquinome treated *S. xylosus* ATCC 700404 cells and non-treated cells] as described in previous study (Xu et al., 2017). iTRAQ analysis was implemented at Shanghai Applied Protein Technology Co., Ltd. (APT, Shanghai, China). iTRAQ analysis was performed as described by Zhao et al. (2015).

### Validation of IGPD Proteomic Analysis by Real-Time PCR Analysis

Selected protein A0A068E9J3 IGPD (*hisB*) was validated at the mRNA level (The *16sRNA* gene was used as internal gene). Target genes’ primers are listed in **Table 1**. Cultures of wild-type *S. xylosus* ATCC700404 strain with 1/2-MIC (0.125 μg/mL) of cefquinome were incubated at 37°C for 24 h. Cells without cefquinome served as control. The cells treated by cefquinome or non-treated were centrifuged at 10,000 × g for 5 min and afterwards treated with an RNASE REMOVER I (Huayueyang Ltd., Beijing, China). An E.Z.N.A.^TM^ Bacterial RNA isolating kit was used. Real-time PCR was performed with an ABI7500 QPCR system (Applied Biosystems, United States) by using SYBR^®^ Premix DimerEraser^TM^ Kit (TaKaRa Biotechnology, Dalian, China). PCR reactions were performed in a total volume of 25 μL containing 12.5 μL of 2 × SYBR^®^ Premix DimerEraser^TM^, 0.5 μL of 50 × ROX Reference Dye II, 1 μL of 10 μmoL/L PCR Forward Primer, 1 μL of 10 μM PCR Reverse Primer, 5 μL of cDNA, and 5 μL of distilled water. Real-time PCR program was: 1 cycle at 95°C for 10 s, and 40 cycles at 95°C for 5 s followed by 55°C for 15 s and 72°C for 30 s.

**Table 1:** The primers used for real-time PCR in the experiment.

Name	Sequence (5′-3′)
*hisB*-F	TACTTCTGTATCACCATT
*hisB*-R	ACTATCTATCTCACTTGC
*16sRNA-*F	CGGGCAATTTGTTTAGCA
*16sRNA*-R	ATTAGGTGGAGCAGGTCA


### Construction of the *hisB* Deletion Mutant Strain

The mutant strain was created using a previously described protocol (Bruckner, 1997) with some modifications. The upstream 1272-bp fragment of the *hisB* gene was amplified from the genomic DNA of *S. xylosus* ATCC700404 using primers *hisB* F- EcoRI and *hisB* F- BamHI. The PCR product was digested and cloned between the EcoRI and BamHI restriction sites of the *E. coli-Staphylococcus* shuttle vector pBT2 (a kind gift from Professor Rui Zhou, Huazhong Agricultural University), resulting in a recombinant plasmid pBT2- *hisB* F. The downstream 1293-bp fragment of the *hisB* gene was amplified using primers *hisB* B- BamHI and *hisB* B- PstI and cloned between the BamHI and PstI restriction sites of pBT2- *hisB* B. The resulted recombinant plasmid was designated as pBT2- *hisB* FB. A 583-bp expression cassette of the erythromycin resistance gene (*ermB*) with BamHI digestion was cloned into the BamHI site of pBT2- *hisB* FB, resulting in a constructed plasmid Δ pBT2 *hisB* for homologous recombination. The shuttle plasmid Δ pBT2 *hisB* was introduced into *S. xylosus* ATCC 700404 by electroporation and selected using chloramphenicol (20 μg/mL) (Bruckner, 1997). The recombinant strains were grown in TSB containing 10 μg/mL erythromycin at 30°C to late-stationary phase. Subsequently, seven passages were performed at 40°C with the exception that erythromycin was omitted in the last passage. Appropriate dilutions of the last passage of culture were spread on TSB agar plates supplemented with 2.5 μg/mL erythromycin and incubated at 37°C overnight. The colonies on these plates were patched onto two TSB agar plates supplemented with either 2.5 μg/mL erythromycin or 20 μg/mL chloramphenicol. Target genes were amplified by PCR with the primers listed in **Table 2**. Identification of *hisB*–deleted mutant strain was performed by PCR analysis.

**Table 2:** Oligonucleotides used in this work.

Oligonucleotide name	Sequence (5′–3′)^a^	Application	
*hisB*_F_up	GAATTCTCATGACAATCCCTCCCAAAAAGTA	Construction of the upstream fragment of the *hisB* gene	
	**(Reference *Staphylococcus xylosus* strain SMQ-121, complete genome)**	
*hisB*_F_down	GGATCCTTGCCCTTATTGATTACGGTTTAGG	Construction of the upstream fragment of the *hisB* gene	
	**(Reference *Staphylococcus xylosus* strain SMQ-121, complete genome)**	
*hisB*_B_up	GGATCCATTGAGACCTCCAAGTTTTAATAAT	Construction of the downstream fragment of the *hisB* gene	
	**(Reference *Staphylococcus xylosus* strain SMQ-121, complete genome)**	
*hisB*_B_down	CTGCAGATTCAAACCGCTTCACCACGAGACT	Construction of the downstream fragment of the *hisB* gene	
	**(Reference *Staphylococcus xylosus* strain SMQ-121, complete genome)**	
*ermB*_up	GGATCCCAGAACAACGTTTACGAATTGGAAC	Construction and checking the *ermB* resistance gene	
	***(Staphylococcus aureus* strain S11 ErmB gene, partial cds)**	
*ermB*_down	GGATCCCTAAATTGTTTACTTTGGCGTGTTT	Construction and checking the *ermB* resistance gene	
	***(Staphylococcus aureus* strain S11 ErmB gene, partial cds)**	
*hisB*-up	GGATCCATGCAAAACAAAAATAGAGTAACTG	Checking of the mutant strain	
	**(Reference *Staphylococcus xylosus* strain SMQ-121, complete genome)**	
*hisB*-down	GTCGACTTAGCTCTGTTCAAAATAACGTGAA	Checking of the mutant strain
	**(Reference *Staphylococcus xylosus* strain SMQ-121, complete genome)**	


**^*a*^Oligonucleotides including the indicated restriction site (underlined).**

### Enzyme Activity Assays

*Staphylococcus xylosus* culture (mid-log growth phase) was supplemented with sub-MICs of cefquinome and cultivated for 24 h at 37°C. Cells supplemented without cefquinome served as control. The cells were collected and centrifuged at 11,000 × *g* for 5 min. After centrifugation, the cells were disrupted by sonication for a total of 15 min at 20% power on ice. Then centrifuged again at 11,000 × *g* for 2 min, and got the cell free extract as the test samples. The activity of the enzyme was determined using a previously described protocol (Martin and Goldberger, 1967) with minor modifications. The reaction mixture consisted of PBS buffer pH 7.4, and cell free extract. The reactions were carried out at 37°C using IGP (Santa Cruz Biotechnology, United States). The reaction was stopped by adding sodium hydroxide at the point with an interval of 30 s. The reaction mixture was then incubated at 37°C for 20 min to convert the product imidazole acetol-phosphate (IAP) into an enolized form, the absorbance of which at 280 nm, was read in a Shimadzu UV spectrophotometer against a blank.

### Determination of Histidine Content

Overnight cultures of *S. xylosus* ATCC 700404 were diluted in sterile TSB (corresponding to 1 × 10^5^ CFU/mL). The diluted overnight cultures were grown (24 h, 37°C) in the presence of sub-MICs of cefquinome. *S. xylosus* ATCC 700404 (Wild-type strain or mutant strain) treated without cefquinome served as a control. The assay was performed using a previously described protocol (Macpherson, 1946). Sample absorbances were read in a Shimadzu UV spectrophotometer against a blank, at 476 nm.

### Statistical Analysis

All experiments were performed in biological triplicates. Statistical comparisons of differences in biofilm formation, iTRAQ analysis, enzyme activity, histidine content and relative gene transcription level were performed using Wilcoxon test (SPSS 11.0.0 statistical software). The data of Real-time PCR were analyzed using repeated measurements in -ΔCt model (Hurtado et al., 2011). For iTRAQ analysis, MS/MS spectra were searched using MASCOT engine (Matrix Science, London, United Kingdom; version 2.2) embedded into Proteome Discoverer 1.3 (Thermo Electron, San Jose, CA, United States) against UniProt database and the decoy database. For protein identification, the following options were used. Peptide mass tolerance = 20 ppm, MS/MS tolerance = 0.1 Da, Enzyme = Trypsin, Missed cleavage = 2, Fixed modification: Carbamidomethyl (C), iTRAQ8plex (K), iTRAQ8plex (N-term), Variable modification: Oxidation (M), FDR ≤ 0.01. The protein had both a fold-change of ratio >1.2 or <0.8 (*p*-value < 0.05). A *p* < 0.05 was considered significant. The values were calculated as the mean of individual experiments in triplicate and compared with those of the control groups.

## Results

### Effect of Cefquinome Against Biofilm Formation *in Vitro*

**FIGURE 1:**
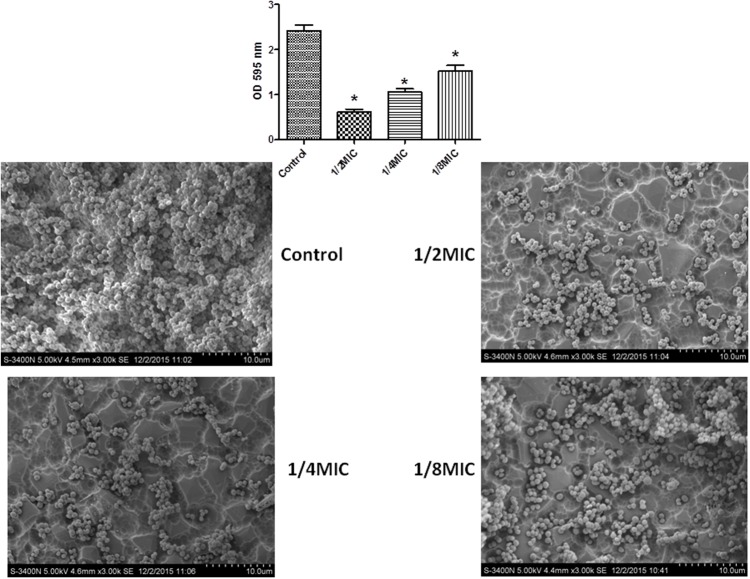
Effect of sub-MICs of cefquinome on biofilm formation by *Staphylococcus xylosus* ATCC700404. The results of the crystal violet staining and scanning electron microscopy (SEM) (Images of the electron microscope) were found at the concentration of each cefquinome concentration. Data are expressed as means ± SDs. Controls refer to the absence of cefquinome. Significantly different (^∗∗^*p* < 0.01, ^∗^*p* < 0.05) compared to untreated control bacteria.

In this study, the MIC value for cefquinome was found to be 0.25 μg/mL for the *S. xylosus* strain of ATCC 700404. Crystal violet staining and SEM (Images of the electron microscope) images were taken for all cefquinome concentrations tested (**Figure 1**). When the culture medium was supplemented with different concentrations of cefquinome, the biofilm biomass formed by *S. xylosus* ATCC700404 was significantly lower in comparison with the control (**Figure 1**). Additionally, for cell cultures without cefquinome, surface of the rough organic membrane was observed to be almost entirely covered by aggregates and micro colonies of *S. xylosus* (**Figure 1**). However, upon the addition of sub-MICs of cefquinome into the culture medium, only a small amount of micro colonies of *S. xylosus* was observed on the rough organic membrane. The effect of cefquinome on inhibiting microcolonies appears to be in a dose-dependent manner. Our results suggest that cefquinome inhibits biofilm formation of *S. xylosus* (**Figure 1**).

### The 1/2-MIC Cefquinome Treated and Non-treated Cells Showed Differently Expression Proteins in Histidine Metabolism Pathway by iTRAQ

**FIGURE 2:**
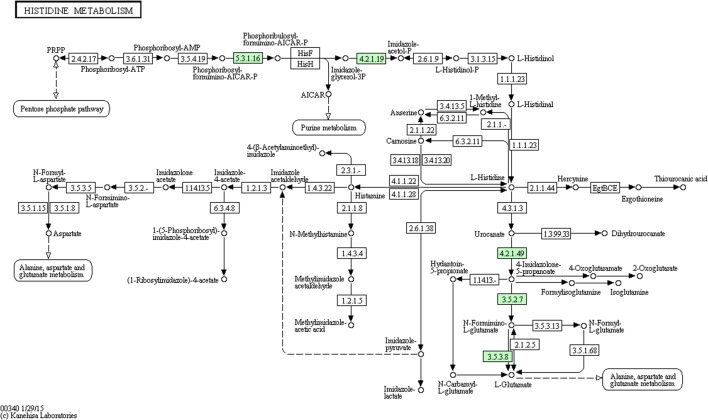
Protein expression profiles during biofilm formation following treatment with cefquinome mapped onto the histidine metabolism pathway. Changes in proteins are marked in green and mapped onto KEGG pathways.

We performed comparative iTRAQ proteomic analysis on cells treated with and without 1/2-MIC. Within the 1768 proteins identified by iTRAQ, 164 proteins were found to be differentially expressed (Details of proteins information see in Supplementary Table S1) in 1/2-MIC cefquinome-treated cells (Proteins with a fold-change of ratio >1.2 or <0.8 (*p*-value < 0.05) were used as selection criteria). We used the KEGG database to analysis these 164 proteins and found five proteins :A0A068E2P9 Imidazolonepropionase (hutI), A0A068E547 Formimidoyl glutamate, A0A068E633 Urocanate hydratase (hutU), A0A068E4P8 1-(5-phosphoribosyl)-5-[(5-phosphoribosylamino)methylideneamino]imidazole-4-carboxamide isomerase (HisA), A0A068E9J3 IGPD (Details of proteins information see in **Table 3**) in histidine metabolism KEGG pathway (**Figure 2**) were altered in the cefquinome-treated cells significantly. Hence we focused on these proteins involved histidine metabolism.

**Table 3:** The histidine metabolism proteins in the KEGG pathway.

Accession	Description	Fold change
A0A068E2P9	Imidazolonepropionase	0.538602283
A0A068E547	Formimidoyl glutamate	0.509939849
A0A068E633	Urocanate hydratase	0.489055704
A0A068E4P8	1-(5-phosphoribosyl)-5-[(5-phosphoribosylamino)methylideneamino] imidazole-4-carboxamide isomerase	0.444199225
A0A068E9J3	Imidazoleglycerol-phosphate dehydratase	0.2518396


### IGPD Is a Potential Target for Cefquinome

**FIGURE 3:**
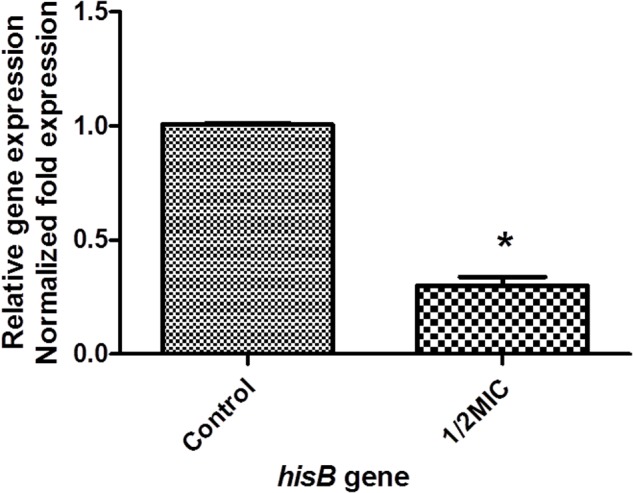
The mRNA expression of *hisB* gene in wild-type *S. xylosus* ATCC700404 strain with 1/2MIC of cefquinome and non-treated. Data are expressed as means ± SDs. The expression was normalized to *16SrRNA*. Controls refer to the absence of cefquinome. Significantly different (^∗^*p* < 0.05) compared to untreated control bacteria.

Our proteomic analyses revealed IGPD expression levels to be drastically reduced. We validated this result at the mRNA level, indicated by the significant down-regulation of *hisB* transcripts (**Figure 3**). We further went on to study the relationship between IGPD and biofilm formation by creating *hisB* knock out mutant strains. PCR analysis indicated efficient knock out of *hisB* gene. A 589-bp *hisB*-specific transcript could only be amplified from the wild-type strains, whereas a 583-bp *ermB*-specific transcript was amplified in the knock out mutant strains confirming *hisB* gene deletion (Supplementary Figure S1).

**FIGURE 4:**
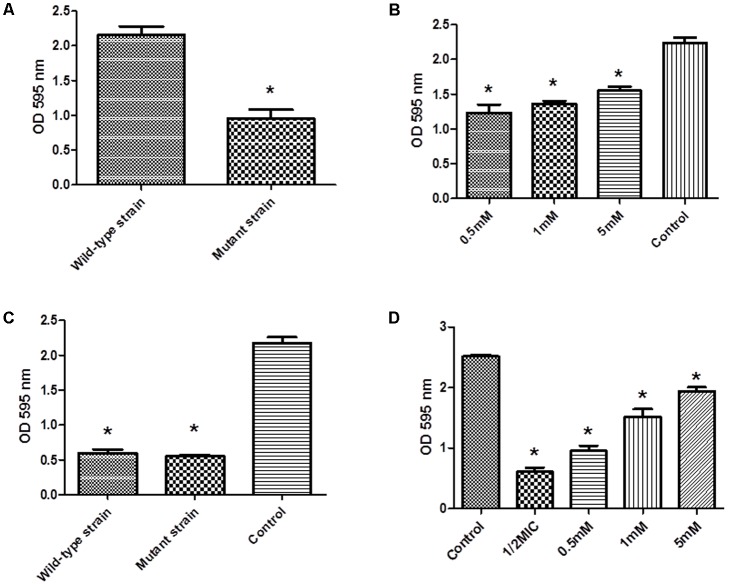
**(A)** Biofilm formation by wild-type *S. xylosus* ATCC700404 strain and mutant (*hisB* gene mutant) *S. xylosus* ATCC700404 strain. **(B)** Biofilm formation by mutant (*hisB* gene mutant) strain grown in the presence of histidine (0.5, 1, and 5 mM). *S. xylosus* ATCC700404 wild-type strain without treatment was served as a control. **(C)** Biofilm formation by wild-type strain and mutant (*hisB* gene mutant) strain grown in the presence of 1/2 minimum inhibitory concentration (MIC) of cefquinome. **(D)** Biofilm formation by wild-type strain treated with 1/2MIC of cefquinome and grown in the presence of histidine (0.5, 1, and 5 mM). *S. xylosus* ATCC700404 wild-type strain without treatment was served as a control. Data are expressed as means ± SDs. Significantly different (^∗^*p* < 0.05, ^∗∗^*p* < 0.01) compared to untreated control bacteria.

We investigated the ability of *hisB* deletion mutant strain in forming biofilms. Our results showed that the ability to form biofilms were severely affected in this mutant strain compare to wild-type strain (**Figure 4A**). Interestingly, when mutant strains were treated with varying amounts of histidine (0.5, 1, and 5 mM), biofilm formation was restored in a concentration-dependent manner (**Figure 4B**). Importantly, when the mutant strains were treated with cefquinome (1/2-MIC), the ability to form biofilms was only weakly affected when compared to the non-treated cells (**Figure 4C**), when wild-type strains in the presence of cefquinome (1/2-MIC) were treated with varying amounts of histidine (0.5, 1, and 5 mM), biofilm formation was also restored in a concentration-dependent manner (**Figure 4D**), suggesting a potential mechanism of action of cefquinome via IGPD.

**FIGURE 5:**
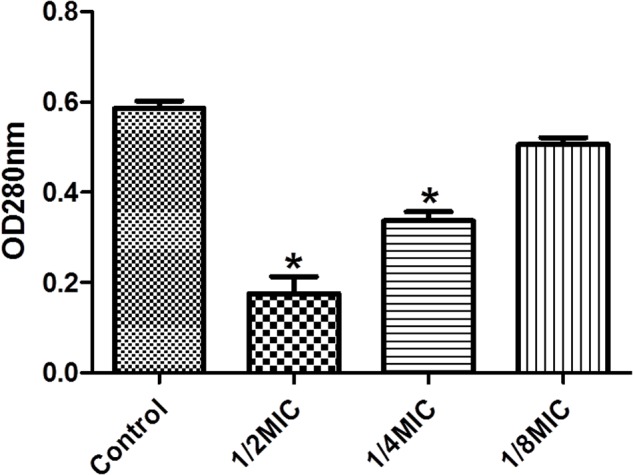
Determination of imidazoleglycerol-phosphate dehydratase (IGPD) activity. Wild-type strain grown in the presence of sub-MICs of cefquinome. *S. xylosus* ATCC700404 was served as a control. Data are expressed as means ± SDs. Significantly different (^∗^*p* < 0.05) compared to untreated control bacteria.

**FIGURE 6:**
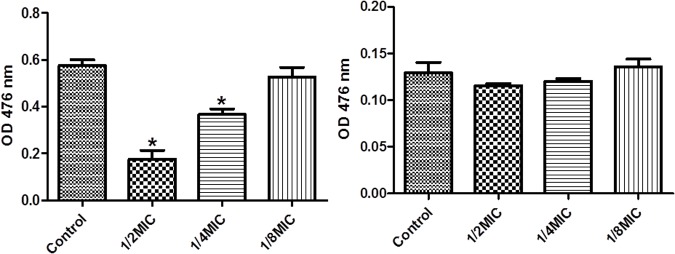
Determination of histidine content. **(A)** *S. xylosus* ATCC700404 wild-type strain grown in the presence of sub-MICs of cefquinome. *S. xylosus* ATCC700404 wild-type strain without cefquinome treated was served as a control. **(B)** *S. xylosus* ATCC700404 mutant strain grown in the presence of sub-MICs of cefquinome. *S. xylosus* ATCC700404 mutant strain without cefquinome treated was served as a control. Data are expressed as means ± SDs. Significantly different (^∗^*p* < 0.05) compared to untreated control bacteria.

We performed enzymatic assays to study the effect of cefquinome on IGPD activity *S. xylosus* ATCC700404 (**Figure 5**). Upon addition of 1/2MIC or 1/4MIC of cefquinome to culture media, IGPD activity of *S. xylosus* ATCC700404 was significantly decreased (*p* < 0.05). However, IGPD activity showed no significant change when the culture medium was supplemented with 1/8MIC of cefquinome, in comparison with the control (*p* > 0.05). A similar result was obtained for the effect of cefquinome on histidine content. We analyzed if histidine content of *S. xylosus* ATCC700404 (Wild-type strain or mutant strain) would be altered when treated with cefquinome. When supplemented with 1/2-MIC and 1/4-MIC of cefquinome in the culture medium, histidine content of *S. xylosus* ATCC700404 (Wild-type strain) showed significant depletion (*p* < 0.05). However, when supplemented with 1/8-MIC of cefquinome in the culture medium, histidine content of *S. xylosus* ATCC700404 (Wild-type strain), in comparison with the control (histidine content of *S. xylosus* ATCC700404 (Wild-type strain) with no cefquinome-treatment as control), was not significantly affected (*p* > 0.05) (**Figure 6A**). Additionally, histidine content of the mutant strain was not significantly affected by cefquinome in comparison with the control (histidine content of *S. xylosus* ATCC700404 (mutant strain) with no cefquinome-treatment as control) (*p* > 0.05) (**Figure 6B**).

## Discussion

*Staphylococcus xylosus* is generally considered as saprophytic and technologically positive in food processing. However, some strains have appeared unexpectedly in bacterial infections in animals (mastitis, dermatitis) and humans (acute pyelonephritis, root canal infection, urinary tract infections) (Won et al., 2002; Cucarella et al., 2004). It has also been shown that *S. xylosus* is resistant to antibiotics treatment (Ugur and Ceylan, 2003). In addition, *S. xylosus* demonstrated the ability to form biofilms, which may impart resistance to host immune system and antibiotics (Parsek and Singh, 2003; Planchon et al., 2006). Owing to the implications of biofilm in playing a role in establishing bacterial resistance, it is vital to understand the mechanism involved in biofilm formation and its prevention. We sought out to investigate this by studying the effect of cefquinome on biofilm formation by *S. xylosus*. Previous studies have indicated that there is a relationship between some antimicrobial agents and biofilm (Majtan et al., 2008; Nucleo et al., 2009; Mishra et al., 2014; Zhao et al., 2015; Wang et al., 2016; Xu et al., 2017). In our study, we found that sub-minimal inhibitory concentrations of cefquinome were sufficient to inhibit biofilm formation, and sub-minimal inhibitory concentrations of cefquinome didn’t affect the growth of *Staphylococcus xylosus* (Detailed information see Supplementary Figure S2).

Biofilm is a process control by various factors, it’s need considerable investigation to understand of biofilm formation better. The iTRAQ technology (Ow et al., 2009) is a useful tool for quantitative proteomics about organism. In this study, we believe that using iTRAQ to identify possible protein targets of cefquinome-mediated inhibition of biofilm formation may provide more systematic information of the two bacterial styles. The differentially expressed proteins may be related to the process by which sub-MIC levels of cefquinome inhibit biofilm formation of *S. xylosus*. We further studied the functional clusters of altered proteins in response to cefquinome using bioinformatics methods. KEGG pathway analysis was performed to better understand the effects of cefquinome on bacterial metabolic pathways. Among them, the histidine metabolism occupied an important proportion. Hence we focussed on proteins involved in the histidine metabolism.

We have previously found that aspirin can inhibit *S. xylosus* biofilm formation and many metabolic pathways may play a role in biofilm formation, e.g., histidine synthesis metabolism being one of them (Xu et al., 2017). In this study, five proteins were clearly down-regulated in the presence of cefquinome. 1-(5-phosphoribosyl)-5-[(5-phosphoribosylamino)methylideneamino] imidazole-4-carboxamide isomerase (HisA) (A0A068E4P8) is involved in step 4 of the pathway that synthesizes L-histidine from 5-phospho-alpha-D-ribose 1-diphosphate, this enzyme is involved in histidine biosynthesis, and has been used as a new target gene to discriminate among the bacteria of the *Burkholderia cepacia* complex species (Papaleo et al., 2010). Formimidoyl glutamate (A0A068E547) is involved in the histidine degradation pathway. Imidazolonepropionase (hutI) (A0A068E2P9) is involved in the pathway that synthesizes L-glutamate from L-histidine. In addition, imidazolonepropionase impacts histidine degradation of mammals and bacteria (Yang et al., 2008). Urocanate hydratase (hutU) (A0A068E633) catalyzes the synthesis of urocanase. Urocanase impacts the L-histidine degradation pathway, and a urocanase mutant strain exhibits reduced biofilm formation compared to wild-type *Acinetobacter baumannii* (Cabral et al., 2011). IGPD is involved in step 6 of the pathway that synthesizes L-histidine from 5-phospho-alpha-D-ribose 1-diphosphate. IGPD is the first enzyme exclusively dedicated to histidine biosynthesis (Dietl et al., 2016). In this study, we have demonstrated that the histidine content was reduced upon the cefquinome treatment in *S. xylosus* cells. However, the histidine content did not show significant changes in the mutant strains suggesting that histidine metabolism might be regulated by cefquinome, thereby imparting an inhibitory effect on the biofilm formation of *S. xylosus*. Based on our findings, IGPD, a key protein of histidine synthesis, was selected as a hypothetical target to screen small molecule drugs that inhibited biofilm formation (Chen et al., 2017). We hypothesized that IGPD might play an important role in inhibiting biofilm formations through the action of cefquinome. Our results (**Figures 3**–**6**) indicate that cefquinome impair the histidine metabolic pathway and the activity of the IGPD. The histidine biosynthetic pathway is only found in lower eukaryotes and prokaryotes but is absent in mammals, which makes these proteins highly attractive targets for the design of new antibacterial drugs (Dietl et al., 2016). Therefore, we propose that IGPD might be targeted by cefquinome, which inhibits biofilm formations of *S. xylosus* upon sub-MICs cefquinome treatment.

## Conclusion

In our study, we found that sub-MICs of cefquinome were sufficient to inhibit biofilm formation. We propose that histidine metabolism might affect biofilm formation. Our study showed a steep down-regulation of an enzyme (A0A068E9J3 IGPD) involved in histidine metabolism pathway, upon cefquinome treatment. Moreover, we demonstrated the important role of IGPD in sub-MICs cefquinome inhibition of biofilm formation of *S. xylosus.* Thus we propose that IGPD could be an attractive target for the design and synthesis of novel anti-biofilm drugs.

## Author Contributions

YL designed the whole experiments. YZ, CX, YY, XX, XL, and QQ performed the experiments. YZ and WD performed the data analysis. GB-O helped to re-analyze the protein data and revised the whole discussion (including grammar errors) of the manuscript.

## Conflict of Interest Statement

The authors declare that the research was conducted in the absence of any commercial or financial relationships that could be construed as a potential conflict of interest.

## Abbreviations

CNS: coagulase-negative *Staphylococcus*
MIC: minimum inhibitory concentration
*S. xylosus*: *Staphylococcus xylosus*
SEM: scanning electron microscopy

## References

[B1] Ahangar M. S. Vyas R. Nasir N. Biswal B. K. (2013). Structures of native, substrate-bound and inhibited forms of *Mycobacterium tuberculosis* imidazoleglycerol-phosphate dehydratase. *Acta Crystallogr. D Biol. Crystallogr.* 69 2461–2467. 10.1107/S0907444913022579 24311587

[B2] Akhaddar A. Elouennass M. Naama O. Boucetta M. (2010). *Staphylococcus xylosus* isolated from an otogenic brain abscess in an adolescent. *Surg. Infect. (Larchmt)* 11 559–561. 10.1089/sur.2010.010 20969474

[B3] Alifano P. Fani R. Lio P. Lazcano A. Bazzicalupo M. Carlomagno M. S. (1996). Histidine biosynthetic pathway and genes: structure, regulation, and evolution. *Microbiol. Rev.* 60 44–69. 8852895 10.1128/mr.60.1.44-69.1996 PMC239417

[B4] Beloin C. Roux A. Ghigo J. M. (2008). *Escherichia coli* biofilms. *Curr. Top. Microbiol. Immunol.* 322 249–289. 10.1007/978-3-540-75418-3_12 18453280 PMC2864707

[B5] Bruckner R. (1997). Gene replacement in *Staphylococcus carnosus* and *Staphylococcus xylosus*. *FEMS Microbiol. Lett.* 151 1–8. 10.1016/S0378-1097(97)00116-X 9198277

[B6] Cabral M. P. Soares N. C. Aranda J. Parreira J. R. Rumbo C. Poza M. (2011). Proteomic and functional analyses reveal a unique lifestyle for *Acinetobacter baumannii* biofilms and a key role for histidine metabolism. *J. Proteome Res.* 10 3399–3417. 10.1021/pr101299j 21612302

[B7] Chen X. F. Wu H. T. Cao Y. Yao X. W. Zhao L. Wang T. Q. (2014). Ion-pairing chromatography on a porous graphitic carbon column coupled with time-of-flight mass spectrometry for targeted and untargeted profiling of amino acid biomarkers involved in *Candida albicans* biofilm formation. *Mol. Biosyst.* 10 74–85. 10.1039/C3MB70240E 24150280

[B8] Chen X. R. Wang X. T. Hao M. Q. Zhou Y. H. Cui W. Q. Xing X. X. (2017). Homology modeling and virtual screening to discover potent inhibitors targeting the imidazole glycerophosphate dehydratase protein in *Staphylococcus xylosus*. *Front. Chem.* 5:98. 10.3389/fchem.2017.00098 29177138 PMC5686052

[B9] Clinical and Laboratory Standards Institute (2003). *Performance Standards for Antimicrobial Susceptibility Testing; Thirteenth Informational Supplement.* Wayne, PA: CLSI.

[B10] Cucarella C. Tormo M. A. Ubeda C. Trotonda M. P. Monzon M. Peris C. (2004). Role of biofilm-associated protein bap in the pathogenesis of bovine *Staphylococcus aureus*. *Infect. Immun.* 72 2177–2185. 10.1128/IAI.72.4.2177-2185.2004 15039341 PMC375157

[B11] Dietl A. M. Amich J. Leal S. Beckmann N. Binder U. Beilhack A. (2016). Histidine biosynthesis plays a crucial role in metal homeostasis and virulence of *Aspergillus fumigatus*. *Virulence* 7 465–476. 10.1080/21505594.2016.1146848 26854126 PMC4871644

[B12] Gaddy J. A. Actis L. A. (2009). Regulation of *Acinetobacter baumannii* biofilm formation. *Future Microbiol.* 4 273–278. 10.2217/fmb.09.5 19327114 PMC2724675

[B13] Hurtado A. Reguant C. Bordons A. Rozés N. (2011). Expression of *Lactobacillus pentosus* B96 bacteriocin genes under saline stress. *Food Microbiol.* 28 1339–1344. 10.1016/j.fm.2011.06.004 21839383

[B14] Kulis-Horn R. K. Persicke M. Kalinowski J. (2014). Histidine biosynthesis, its regulation and biotechnological application in *Corynebacterium glutamicum*. *Microb. Biotechnol.* 7 5–25. 10.1111/1751-7915.12055 23617600 PMC3896937

[B15] Latasa C. Solano C. Penades J. R. Lasa I. (2006). Biofilm-associated proteins. *C. R. Biol.* 329 849–857. 10.1016/j.crvi.2006.07.008 17067927

[B16] Macpherson H. T. (1946). The basic amino-acid content of proteins. *Biochem. J.* 40 470–481. 10.1042/bj0400470 PMC1258383 16748039

[B17] Majtan J. Majtanova L. Xu M. Majtan V. (2008). *In vitro* effect of subinhibitory concentrations of antibiotics on biofilm formation by clinical strains of *Salmonella enterica* serovar Typhimurium isolated in Slovakia. *J. Appl. Microbiol.* 104 1294–1301. 10.1111/j.1365-2672.2007.03653.x 18028358

[B18] Martin R. G. Goldberger R. F. (1967). Imidazolylacetolphosphate: L-glutamate aminotransferase. Purification and physical properties. *J. Biol. Chem.* 242 1168–1174. 5337155

[B19] Mishra N. N. Ali S. Shukla P. K. (2014). Arachidonic acid affects biofilm formation and PGE2 level in *Candida albicans* and non-*albicans* species in presence of subinhibitory concentration of fluconazole and terbinafine. *Braz. J. Infect. Dis.* 18 287–293. 10.1016/j.bjid.2013.09.006 24389279 PMC9427476

[B20] Nucleo E. Steffanoni L. Fugazza G. Migliavacca R. Giacobone E. Navarra A. (2009). Growth in glucose-based medium and exposure to subinhibitory concentrations of imipenem induce biofilm formation in a multidrug-resistant clinical isolate of *Acinetobacter baumannii*. *BMC Microbiol.* 9:270. 10.1186/1471-2180-9-270 20028528 PMC2804601

[B21] Ow S. Y. Salim M. Noirel J. Evans C. Rehman I. Wright P. C. (2009). iTRAQ underestimation in simple and complex mixtures: “The Good, the Bad and the Ugly”. *J. Proteome Res.* 8 5347–5355. 10.1021/pr900634c 19754192

[B22] Papaleo M. C. Perrin E. Maida I. Fondi M. Fani R. Vandamme P. (2010). Identification of species of the *Burkholderia cepacia* complex by sequence analysis of the *hisA* gene. *J. Med. Microbiol.* 59 1163–1170. 10.1099/jmm.0.019844-0 20651037

[B23] Parsek M. R. Singh P. K. (2003). Bacterial biofilms: an emerging link to disease pathogenesis. *Annu. Rev. Microbiol.* 57 677–701. 10.1146/annurev.micro.57.030502.090720 14527295

[B24] Planchon S. Desvaux M. Chafsey I. Chambon C. Leroy S. Hebraud M. (2009). Comparative subproteome analyses of planktonic and sessile *Staphylococcus xylosus* C2a: new insight in cell physiology of a coagulase-negative *Staphylococcus* in biofilm. *J. Proteome Res.* 8 1797–1809. 10.1021/pr8004056 19253936

[B25] Planchon S. Gaillard-Martinie B. Dordet-Frisoni E. Bellon-Fontaine M. N. Leroy S. Labadie J. (2006). Formation of biofilm by *Staphylococcus xylosus*. *Int. J. Food Microbiol.* 109 88–96. 10.1016/j.ijfoodmicro.2006.01.016 16503066

[B26] Rumi M. V. Huguet M. J. Bentancor A. B. Gentilini E. R. (2013). The icaA gene in staphylococci from bovine mastitis. *J. Infect. Dev. Countr.* 7 556–560. 10.3855/jidc.2670 23857391

[B27] Sauer K. (2003). The genomics and proteomics of biofilm formation. *Genome Biol.* 4:219. 10.1186/gb-2003-4-6-219 12801407 PMC193612

[B28] Stepanovic S. Vukovic D. Dakic I. Savic B. Svabic-Vlahovic M. (2000). A modified microtiter-plate test for quantification of staphylococcal biofilm formation. *J. Microbiol. Methods* 40 175–179. 10.1016/S0167-7012(00)00122-6 10699673

[B29] Sun L. N. Chen H. R. Lin W. X. Lin X. M. (2017). Quantitative proteomic analysis of *Edwardsiella tarda* in response to oxytetracycline stress in biofilm. *J. Proteomics* 150 141–148. 10.1016/j.jprot.2016.09.006 27638425

[B30] Swinkels J. M. Lam T. Green M. J. Bradley A. J. (2013). Effect of extended cefquinome treatment on clinical persistence or recurrence of environmental clinical mastitis. *Vet. J.* 197 682–687. 10.1016/j.tvjl.2013.03.010 23702283

[B31] Tan X. J. Liu L. Liu S. L. Yang D. T. Liu Y. K. Yang S. (2014). Genome of *Staphylococcus xylosus* and comparison with *S. aureus* and *S. epidermidis.* *J. Genet. Genomics* 41 413–416. 10.1016/j.jgg.2014.03.007 25064680

[B32] Ugur A. Ceylan O. (2003). Occurrence of resistance to antibiotics, metals, and plasmids in clinical strains of *Staphylococcus* spp. *Arch. Med. Res.* 34 130–136. 10.1016/S0188-4409(03)00006-7 12700009

[B33] Wang S. Yang Y. B. Zhao Y. L. Zhao H. H. Bai J. W. Chen J. Q. (2016). Sub-MIC tylosin inhibits *Streptococcus suis* biofilm formation and results in differential protein expression. *Front. Microbiol.* 7:384. 10.3389/fmicb.2016.00384 27065957 PMC4811924

[B34] Wang Y. Yi L. Wu Z. F. Shao J. Liu G. J. Fan H. J. (2012). Comparative proteomic analysis of *Streptococcus suis* biofilms and planktonic cells that identified biofilm infection-related immunogenic proteins. *PLoS One* 7:e33371. 10.1371/journal.pone.0033371 22514606 PMC3326019

[B35] Won Y. S. Kwon H. J. Oh G. T. Kim B. H. Lee C. H. Park Y. H. (2002). Identification of *Staphylococcus xylosus* isolated from C57BL/6J-*Nos2^tm1Lau^* mice with dermatitis. *Microbiol. Immunol.* 46 629–632. 10.1111/j.1348-0421.2002.tb02744.x 12437030

[B36] Xu C. G. Yang Y. B. Zhou Y. H. Hao M. Q. Ren Y. Z. Wang X. T. (2017). Comparative proteomic analysis provides insight into the key proteins as possible targets involved in aspirin inhibiting biofilm formation of *Staphylococcus xylosus*. *Front. Pharmacol.* 8:543. 10.3389/fphar.2017.00543 28871227 PMC5566577

[B37] Yang F. F. Chu W. S. Yu M. J. Wang Y. Ma S. X. Dong Y. H. (2008). Local structure investigation of the active site of the imidazolonepropionase from *Bacillus subtilis* by XANES spectroscopy and *ab initio* calculations. *J. Synchrotron Radiat.* 15 129–133. 10.1107/S0909049507064126 18296777

[B38] Zeidan M. B. Zara G. Viti C. Decorosi F. Mannazzu I. Budroni M. (2014). L-Histidine inhibits biofilm formation and *FLO11*-associated phenotypes in *Saccharomyces cerevisiae* flor yeasts. *PLoS One* 9:e112141. 10.1371/journal.pone.0112141 25369456 PMC4219837

[B39] Zhao Y. L. Zhou Y. H. Chen J. Q. Huang Q. Y. Han Q. Liu B. (2015). Quantitative proteomic analysis of sub-MIC erythromycin inhibiting biofilm formation of *S. suis* in vitro. *J. Proteomics* 116 1–14. 10.1016/j.jprot.2014.12.019 25579403

