# Supplementary material for: Histidine Metabolism and IGPD Play a Key Role in Cefquinome Inhibiting Biofilm Formation of Staphylococcus xylosus
Source: Front Microbiol. 2018 Apr 5;9:665. doi: 10.3389/fmicb.2018.00665 (PMC5896262; doi:10.3389/fmicb.2018.00665)
Supplement: Supplementary file 1 [file Table_1.DOCX]

**iTRAQ Identification of Differentially Expressed Proteins with 1/2-MIC cefquinome (*p*<0.05)**

| **Accession** | **Description** | **Fold change** |
| --- | --- | --- |
| **A0A068E269** | **Uncharacterized protein** | **4.8092068** |
| **A0A060MFJ4** | **Uncharacterized protein** | **3.005911806** |
| **A0A068E690** | **L-lactate dehydrogenase** | **2.520538266** |
| **A0A068E914** | **Putative peptidase** | **2.224639934** |
| **A0A090LYG3** | **Nitroreductase family protein** | **1.979061294** |
| **K8DVZ1** | **Uncharacterized protein** | **1.852201992** |
| **A0A068E5T2** | **Putative permease of the major facilitator superfamily** | **1.78758089** |
| **A0A060MLZ9** | **Putative DUF1027 domain protein** | **1.773028336** |
| **A0A068ED22** | **ComK regulator** | **1.755464112** |
| **A0A068E841** | **Acetyl-CoA carboxylase, biotin carboxyl carrier protein** | **1.748636284** |
| **A0A060MDK6** | **50S ribosomal protein L35** | **1.747109075** |
| **A0A068EE50** | **Uncharacterized protein** | **1.737509278** |
| **A0A068E8A4** | **Rrf2-linked NADH-flavin reductase** | **1.730325132** |
| **A0A060MEP0** | **50S ribosomal protein L36** | **1.690995038** |
| **A0A060MER3** | **30S ribosomal protein S14 type Z** | **1.688094484** |
| **A0A068E5L4** | **GTP pyrophosphokinase** | **1.659167552** |
| **A0A060MQ60** | **Fructose-bisphosphate aldolase** | **1.627101208** |
| **A0A068E9Q9** | **Phosphoglycerate kinase** | **1.620598522** |
| **A0A060MBV1** | **UPF0413 protein** | **1.61491996** |
| **A0A068E6Z9** | **Diphosphomevalonate decarboxylase** | **1.601083365** |
| **A0A068ECE4** | **GMP reductase** | **1.583250755** |
| **A0A090M350** | **Flavohemoprotein** | **1.582687861** |
| **A0A060MMS2** | **50S ribosomal protein L28** | **1.574892864** |
| **A0A060MHF0** | **30S ribosomal protein S21** | **1.568997949** |
| **A0A068E857** | **Flavohemoprotein** | **1.568875228** |
| **A0A068E9W7** | **6-carboxy-5,6,7,8-tetrahydropterin synthase** | **1.559541666** |
| **A0A060MSF6** | **Putative amino acid transporter** | **1.536646898** |
| **A0A090K3S3** | **2,3-bisphosphoglycerate-independent phosphoglycerate mutase** | **1.535514992** |
| **A0A068E538** | **Putative glycosyl/glycerophosphate transferases** | **1.529646248** |
| **A0A090K6V7** | **Phage integrase** | **1.527814812** |
| **A0A090K9H4** | **DNA polymerase X family protein** | **1.527736209** |
| **A0A090KA05** | **Argininosuccinate synthase** | **1.525199639** |
| **A0A068E7G2** | **Glycerol-3-phosphate dehydrogenase** | **1.51478612** |
| **A0A068E703** | **Branched-chain alpha-keto acid dehydrogenase** | **1.511172163** |
| **A0A060MIP0** | **Cobalt-zinc-cadmium resistance protein** | **1.508055514** |
| **A0A060MN37** | **Glycerol-3-phosphate acyltransferase** | **1.503753206** |
| **A7KJI9** | **Enolase OS=Staphylococcus xylosus** | **1.500333757** |
| **A0A060MQF1** | **Putative transcriptional regulator** | **1.497667195** |
| **A0A068E3Q1** | **UPF0176 protein SXYL_00120** | **1.494859531** |
| **A0A068EC44** | **Putative carboxy-terminal processing proteinase ctpA** | **1.488818846** |
| **A0A060ME04** | **Uncharacterized protein** | **1.480844359** |
| **A0A090K0Y1** | **Similar to phage-related protein** | **1.478824657** |
| **A0A090K5X6** | **GMP synthase [glutamine-hydrolyzing]** | **1.473342169** |
| **A0A068E9Z2** | **Uncharacterized protein** | **1.472286132** |
| **A0A090K3E4** | **ATP-dependent protease ATPase subunit HslU** | **1.468311563** |
| **A0A090KAT8** | **Putative membrane protein** | **1.465607457** |
| **A0A060MLT3** | **Triosephosphate isomerase** | **1.458910496** |
| **A0A068EAX9** | **Allantoate amidohydrolase** | **1.45850745** |
| **A0A060MDC8** | **30S ribosomal protein S20** | **1.45704103** |
| **A0A068E6G2** | **Protoheme IX farnesyltransferase** | **1.453177046** |
| **A0A060MN39** | **Thymidylate kinase** | **1.450129221** |
| **A0A068E4G1** | **Pyruvate oxidase, CidC** | **1.449102852** |
| **A0A060ML60** | **Phospho-N-acetylmuramoyl-pentapeptide-transferase** | **1.447652425** |
| **A0A068E4E9** | **Transcription regulator** | **1.44742104** |
| **A0A068E327** | **Zinc ABC transporter, periplasmic-binding protein ZnuA** | **1.445458936** |
| **A0A068E2F4** | **Phage infection protein** | **1.43644108** |
| **A0A068E7R4** | **Transcriptional regulator** | **1.436232064** |
| **A0A068E3B8** | **Ribokinase** | **1.434005193** |
| **A0A068E497** | **Putative exported protein** | **1.430223721** |
| **A0A068E5R8** | **50S ribosomal protein L32** | **1.427639877** |
| **A0A068E2N0** | **TetR family regulatory protein of MDR cluster** | **1.426650101** |
| **P51184** | **PTS system sucrose-specific EIIBC component]** | **1.426254121** |
| **A0A060MEN2** | **30S ribosomal protein S9** | **1.422266116** |
| **A0A090K3D2** | **3-methyl-2-oxobutanoate hydroxymethyltransferase** | **1.417901275** |
| **C6ZDG3** | **AlG2-like protein** | **1.415867244** |
| **A0A060MN61** | **UPF0435 protein SXYL_00956** | **0.546412271** |
| **A0A068E821** | **Methylenetetrahydrofolate reductase** | **0.546321196** |
| **A0A060MK38** | **LysR family transcriptional regulator** | **0.545147908** |
| **A0A068E6D5** | **Succinate dehydrogenase flavoprotein subunit** | **0.544057029** |
| **A0A068E2S8** | **L-lactate dehydrogenase** | **0.542551268** |
| **A0A068E7W5** | **Succinate dehydrogenase iron-sulfur protein** | **0.542230339** |
| **A0A060MC68** | **UPF0358 protein SXYL_01823** | **0.541175459** |
| **A0A060MJN5** | **3-oxoacyl-[acyl-carrier protein] reductase** | **0.540459938** |
| **A0A060MBM8** | **Glycine cleavage system H protein** | **0.540213961** |
| **A0A090K041** | **Pyrrolidone-carboxylate peptidase** | **0.540079273** |
| **A0A068E2P9** | **Imidazolonepropionase** | **0.538602283** |
| **A0A068E4K5** | **Acetyltransferase (GNAT) family protein** | **0.537794502** |
| **A0A068E7D5** | **Threonine synthase** | **0.537499052** |
| **A0A060MNR8** | **Uncharacterized protein** | **0.537481706** |
| **A0A068E8Z3** | **Manganese ABC transporter, periplasmic-binding protein SitA** | **0.53666482** |
| **A0A068E9J8** | **D-alanine--poly(phosphoribitol) ligase subunit 1** | **0.536355768** |
| **A0A068EDB8** | **Bifunctional autolysin Atl** | **0.535957438** |
| **A0A068EDJ4** | **ABC transporter, permease protein** | **0.533414308** |
| **A0A068EDF8** | **Ornithine aminotransferase** | **0.531704261** |
| **A0A068E516** | **Uncharacterized protein** | **0.531653942** |
| **A0A068E275** | **N-acetylneuraminate lyase** | **0.531263965** |
| **A0A060MF57** | **Uncharacterized protein** | **0.53044803** |
| **A0A068E3I3** | **Beta-glucoside bgl operon antiterminator, BglG family** | **0.529975011** |
| **A0A068E4Z7** | **Biotinyl-lipoyl attachment domain protein, GcvH-like protein** | **0.529272256** |
| **A0A090JY89** | **Probable transglycosylase sceD 2** | **0.528938144** |
| **A0A068E7H1** | **Acetoin utilization protein AcuA** | **0.528064214** |
| **A0A068E6R2** | **Cobalamin-independent methionine synthase II** | **0.524900083** |
| **A0A068E8C4** | **Oligopeptide transport ATP-binding protein OppD** | **0.518793767** |
| **A0A060MNE6** | **L-threonine dehydratase** | **0.510795883** |
| **A0A068E547** | **Formimidoylglutamase** | **0.509939849** |
| **A0A068E9E8** | **Aldehyde dehydrogenase A** | **0.505935939** |
| **A0A060MRA8** | **Cold shock protein** | **0.498798633** |
| **A0A060MDH7** | **UPF0337 protein** | **0.498131708** |
| **A0A068E1P6** | **Transcriptional regulator** | **0.494506157** |
| **A0A068E7A3** | **Mannonate dehydratase** | **0.492180612** |
| **A0A068EB44** | **6,7-dimethyl-8-ribityllumazine synthase** | **0.489872913** |
| **A0A068E633** | **Urocanate hydratase** | **0.489055704** |
| **A0A068E8D8** | **Putative membrane protein** | **0.481304818** |
| **A0A060MLT7** | **Preprotein translocase subunit SecG** | **0.481217316** |
| **A0A060MBN2** | **Lipoprotein** | **0.478171255** |
| **A0A090JZD9** | **Pseudouridine-5'-phosphate glycosidase** | **0.472851117** |
| **A0A090M2A6** | **Homoserine dehydrogenase** | **0.471439293** |
| **A0A068E1W8** | **Gluconate permease** | **0.468457485** |
| **A0A068E2Y3** | **Putative permease of the major facilitator superfamily** | **0.467983751** |
| **A0A068E9C9** | **2-dehydro-3-deoxyphosphogluconate aldolase** | **0.463763216** |
| **A0A068E540** | **Biotin carboxylase** | **0.456367945** |
| **A0A068EAA0** | **Uncharacterized protein** | **0.451908842** |
| **A0A068E3H6** | **3-isopropylmalate dehydrogenase** | **0.446711395** |
| **A0A068E7B7** | **Uncharacterized protein** | **0.445693214** |
| **A0A068E4P8** | **1-(5-phosphoribosyl)-5-[(5-phosphoribosylamino)methylideneamino]**  **imidazole-4-carboxamide isomerase** | **0.444199225** |
| **A0A068E3T6** | **FmtB protein** | **0.441269938** |
| **A0A068E8Q9** | **Ribonucleotide reductase of class III (Anaerobic), large subunit** | **0.438809767** |
| **A0A090K9X7** | **Oligopeptide ABC transporter, periplasmic oligopeptide-binding protein OppA** | **0.435728884** |
| **A0A068E4N6** | **Beta-N-acetylhexosaminidase** | **0.4347521** |
| **A0A060MB56** | **Na(+) H(+) antiporter subunit E** | **0.433588595** |
| **A0A068E2L5** | **Glycerate kinase** | **0.432369032** |
| **A0A060MLJ0** | **Aspartokinase** | **0.431054082** |
| **A0A090K4Y0** | **Putative NCAIR mutase** | **0.428978496** |
| **A0A068EE66** | **Inosose isomerase** | **0.423979827** |
| **A0A090K3G9** | **Putative pyruvate, phosphate dikinase regulatory protein** | **0.42019911** |
| **A0A060MHP5** | **Acetolactate synthase small subunit** | **0.417588802** |
| **A0A068E8J7** | **Oxidoreductase, short chain dehydrogenase/reductase family** | **0.40858341** |
| **A0A068E407** | **Acetyl-coenzyme A synthetase** | **0.40558159** |
| **A0A090K0U9** | **Sodium:dicarboxylate transporter** | **0.400719494** |
| **A0A090K5Y4** | **Proline dehydrogenase** | **0.398150538** |
| **A0A090K0Y5** | **Putative phage protein** | **0.396557625** |
| **A0A068E526** | **Gamma-aminobutyrate:alpha-ketoglutarate aminotransferase** | **0.39443196** |
| **A0A090K7G3** | **Phosphoenolpyruvate carboxykinase [ATP]** | **0.392066588** |
| **A7IY64** | **Probable transglycosylase IsaA** | **0.384882108** |
| **A0A090K3I0** | **Putative Succinate-semialdehyde dehydrogenase** | **0.381946003** |
| **A0A060MRD2** | **1-pyrroline-5-carboxylate dehydrogenase** | **0.381802976** |
| **A0A060MCL3** | **Uncharacterized protein** | **0.380872727** |
| **A0A068ECI5** | **Glycerol uptake facilitator protein** | **0.380493758** |
| **A0A090JZ46** | **390-kDa surface protein** | **0.378112956** |
| **A0A068E4G9** | **Acetylornithine deacetylase** | **0.376096607** |
| **A0A060MH08** | **Glyceraldehyde-3-phosphate dehydrogenase** | **0.375621801** |
| **A0A090KBH6** | **Truncated lipase GehM** | **0.371981225** |
| **A0A068E7D1** | **Catalase** | **0.370258757** |
| **A0A090JZ91** | **Pyruvate phosphate dikinase** | **0.369394024** |
| **A0A068E533** | **Tautomerase** | **0.360836398** |
| **A0A090LY94** | **Gluconate permease** | **0.357374889** |
| **A0A068EF38** | **Alpha-galactosidase** | **0.357087163** |
| **A0A068E295** | **Glucarate transporter** | **0.343797271** |
| **A0A090JZE5** | **Putative PTS multidomain regulator** | **0.328061988** |
| **A0A068E9F7** | **Sodium/proline symporter** | **0.327499522** |
| **A0A090K528** | **Similar to quorum-quenching N-acyl homoserine lactonase** | **0.320541659** |
| **A0A068E8L1** | **Glutamine synthetase** | **0.315735196** |
| **A0A068E4J3** | **Putative sialic acid transporter** | **0.312374633** |
| **A0A068E3A0** | **Arginase** | **0.302959725** |
| **A0A068E420** | **PTS system, mannitol-specific IIB component** | **0.29698058** |
| **A0A060MF11** | **Oligopeptide transport system permease protein OppB** | **0.294490057** |
| **A0A060MLC9** | **Uncharacterized protein** | **0.290750324** |
| **A0A068E276** | **Uncharacterized protein** | **0.288760083** |
| **A0A068E2L0** | **UPF0272 protein SXYLSMQ121_0200** | **0.284491797** |
| **A0A068E9J3** | **Imidazoleglycerol-phosphate dehydratase** | **0.2518396** |
| **A0A068E843** | **Hydrolase (HAD superfamily)** | **0.242321395** |
| **A0A068E6R7** | **Sodium/alanine symporter family protein** | **0.226656536** |
| **A0A068E3Z3** | **PhnB protein** | **0.201738296** |
| **A0A060MNG4** | **Nucleoside diphosphate kinase** | **0.17849896** |
