# Supplementary material for: Histidine Metabolism and IGPD Play a Key Role in Cefquinome Inhibiting Biofilm Formation of Staphylococcus xylosus
Source: Front Microbiol. 2018 Apr 5;9:665. doi: 10.3389/fmicb.2018.00665 (PMC5896262; doi:10.3389/fmicb.2018.00665)
Supplement: Supplementary file 2 [file Presentation_1.pdf]

Figure S1.

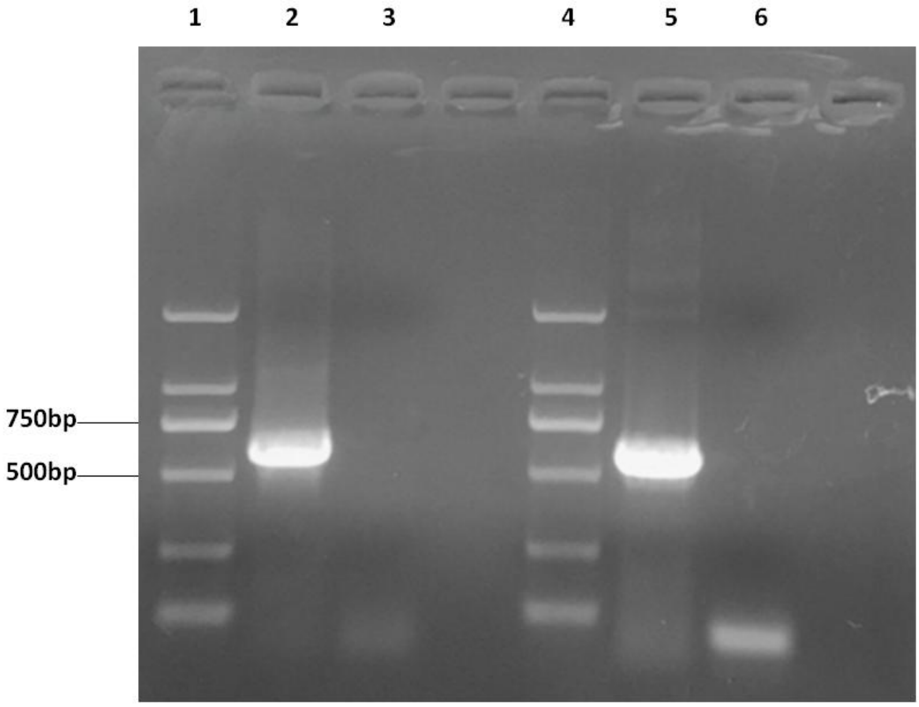

Identification of *hisB*–deleted mutant strain by PCR analysis. Line 1,4 Marker DL2000, line 3,6 mutant strain, line 2,5 wild-type strain, line 2,3 *ermB* gene, line 5,6 *hisB* gene.

Determination of growth inhibition activity of cefquinome

The growth rates of *S. xylosus* ATCC700404 treated with sub-MIC of cefquinome were analyzed. Briefly, the overnight strains were grown in 5 ml TSB medium at 37 ℃. Then, dilute the cultures of cells at the concentration to $1x10^5$ CFU / ml and added sub-MIC of cefquinome incubated at 37 ^{o}C for 24 h. The samples were taken every hour for measuring OD 595 nm.

Figure S2.

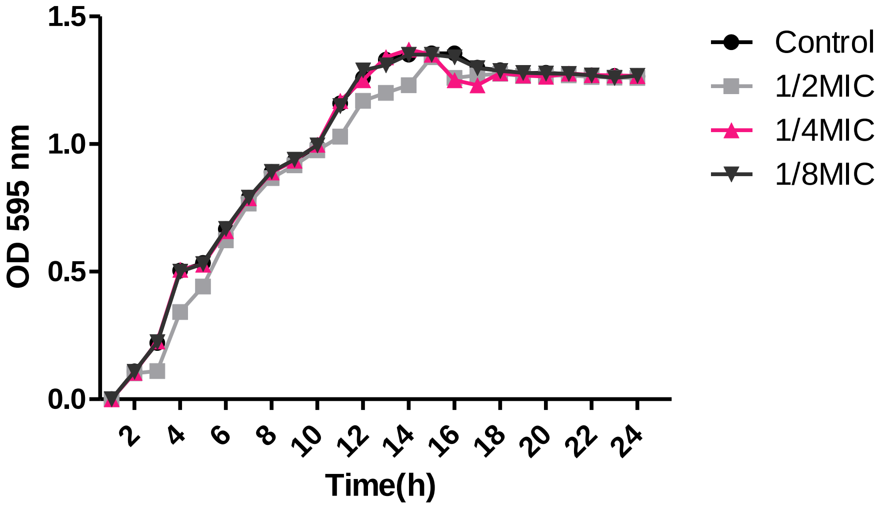

From this Figure, we found that the growth was not changed (p>0.05) by 1/2, 1/4 and 1/8 MIC of cefquinome.
